# Local Government Debt and Green Total Factor Productivity—Empirical Evidence from Chinese Cities

**DOI:** 10.3390/ijerph191912425

**Published:** 2022-09-29

**Authors:** Ke Mao, Pierre Failler

**Affiliations:** 1 Business School, Xiangtan University, Xiangtan 411105, China; 2 Business School, Hunan Institute of Technology, Hengyang 421002, China; 3 Economics and Finance Group, Portsmouth Business School, University of Portsmouth, Portsmouth PO1 3DE, UK

**Keywords:** local government debt (LGD), green total factor productivity (GTFP), green innovation, financial market development

## Abstract

In recent years, the expansion of local government debt (LGD) in China has caused widespread concern. Enhancing green total factor productivity (GTFP) is an important way to coordinate resources, environment, and regional development and is an important indicator to realize the transformation of green economic development. Scientific assessment of the impact of LGD on GTFP helps promote the transformation of green economic development. This paper selects sample data from 271 cities in China from 2010 to 2019 and empirically investigates the mechanisms of LGD, green innovation, and financial market development on GTFP. The results show that (1) LGD expansion significantly suppresses GTFP in China; (2) green innovation mediates between the two, and LGD suppresses GTFP by reducing the level of green innovation; and (3) financial market development can mitigate the negative impact of LGD on urban GTFP. Therefore, the governance of LGD should be strengthened, the financial market environment should be optimized, the distortion of financial resources should be corrected, and innovative financing modes such as green finance and green credit should be encouraged to enhance GTFP.

## 1. Introduction

China has recently experienced two pressures that have drawn significant international attention: slowed economic development and a substantial increase in local government debt (LGD) [1]. The Chinese government executed a CNY 4 trillion (equivalent to EUR 586 billion) fiscal stimulus plan starting during the financial crisis of 2008, promoting economic recovery while causing a sharp increase in public debt [2]. According to the National Audit Office of China, in 2013, the LGD balance was CNY 17.89 trillion (equivalent to EUR 2.62 trillion), while the central government’s debt balance was CNY 12.38 trillion (equivalent to EUR 1.81 trillion), with the scale of LGD exceeding that of the central government debt. Currently, improving green total factor productivity (GTFP) is a key step toward achieving sustainable economic development in China, which is in the midst of a crucial transition from the country’s previous crude development to green and sustainable development [3]. Therefore, a scientific assessment of the impact of LGD expansion on GTFP can not only provide a basis for the governance of public debt but also clarify potential obstacle factors for promoting green economic development.

The existing studies on public debt have mainly focused on the relationship between government debt and economic variables such as economic growth [4,5,6,7], capital allocation efficiency [8,9], inflation [10,11], market interest rates [12], and financial risk [13], with less research on environmental effects. Fodha and Seegmuller (2014) [14] constructed a two-period overlapping generations theoretical model demonstrating how increased public debt used to fund environmental-protection initiatives can enhance environmental quality. Clootens (2017) [15] discovered that public debt has a favorable impact on environmental quality up to a particular debt level and turns negative if the ratio is exceeded. Zhang and Zhao (2018) [16] discovered that the increased quantity of government debt causes local governments to weaken the rigor of environmental rules to draw private investment, to alleviate debt pressure, and to collect funding for debt servicing, which worsens environmental pollution. Carratù et al. (2019) [17], based on 24 European countries’ panel data from 1996 to 2015, empirically found a nonlinear effect of public debt on air pollution. Chen et al. (2022) [18], based on Chinese provincial panel data, empirically found that rising LGD levels exacerbate corporate financing constraints, which in turn undermine corporate GI capacity.

Although there is a lot of literature on the relationship between public debt and economic growth, and environmental pollution, on the one hand, there is still a research gap on the impact of public debt on GTFP; on the other hand, most of the existing literature analyzes the impact of public debt at the national and provincial levels, and few studies analyze the impact of public debt at the city level. Therefore, this paper explores the impact of LGD on GTFP based on urban panel data from 2010 to 2019 using a fixed-effects panel model. The results show that LGD expansion significantly inhibits GTFP in China.

The main contributions of this paper are as follows: first, the existing studies mainly pay close attention to the economic costs of public debt, while limited studies on the environmental effects of public debt only study the effects of environmental quality and pollution. This paper considers the combined economic and environmental effects of public debt and examines how LGD affects GTFP, which enriches the relevant studies. Second, most existing studies have been conducted at the national and provincial levels, ignoring the differences between individual cities; this paper provides a more detailed analysis based on the empirical study of panel data of 271 cities in China. Third, most of the existing literature studies the direct mechanism of LGD on environmental pollution, with little attention to the important mediating mechanism of green innovation (GI). This paper incorporates the level of GI into the analytical framework, reveals how LGD influences GTFP, and conducts a mechanism test, which helps to find out how public debt affects green development. Finally, the level of financial market development (FMD) is introduced as a moderating variable to show that FMD can effectively lessen the negative influence of LGD on GTFP and provide new ideas for the government to manage public debt.

The paper is structured as follows: Section 2 presents the theoretical analysis and research hypotheses of the paper; Section 3 presents empirical model and methodology; Section 4 presents definitions of variable and data Sources; Section 5 presents the empirical results and analysis; and Section 6 presents the research conclusions and recommendations.

## 2. Theoretical Analysis and Research Hypotheses

China passed a tax-sharing reform in 1994 that stripped local governments of their ability to levy taxes, causing a sharp decrease in local government revenues [19,20]. Since the promotion of government officials is related to the local economic performance of their tenure [21], in the context of facing fiscal pressure, officials who are eager to create promotion performance during their tenure will increase public spending to promote economic growth and achieve economic growth goals by raising debt. This development model of increasing public capital expenditure through large-scale debt by local governments can promote short-term economic growth. Nevertheless, it can inhibit long-term economic growth [9,22], which is not conducive to sustainable economic development. Moreover, LGD expansion can reduce the allocation efficiency of capital markets [8]; cause a capital mismatch between industries, firms, and regions [23]; and inhibit GTFP.

From the perspective of debt repayment, excessive expansion of LGD will cause increased pressure on LGD service. Making local officials increase fiscal revenue, on the one hand, may relax entry restrictions on highly polluting enterprises, attracting more polluting enterprises to invest locally; on the other hand, they may relax environmental regulation of local enterprises, lowering their environmental treatment costs and increasing their profits, but will exacerbate the local environmental pollution problems [24]. In addition, land lease income is an important source of debt service and interest payment for local governments [25,26], and the resulting large-scale industrial land development will lead to increased pollution emissions, which will intensify environmental pollution and is detrimental to the green development. Based on this, the following hypothesis is proposed.

**H1.**  *LGD can significantly inhibit GTFP.*

The level of GI is an important driver of GTFP growth [27]. However, GI projects are characterized by long cycles, costs, and high uncertainty [28], and financing constraints are the primary challenge inhibiting GI activities [29]. LGD can exacerbate the problem of financing constraints for GI projects. First, LGD financing can reduce the number of funds available to support GI activities. In the credit market, the government owns high-quality assets such as land ownership, which is more favored by bank collateral [30], and more importantly, the government may preferentially obtain credit funds from banks through administrative intervention [31]. Therefore, banks will prioritize local government financing and squeeze out the financing demand for GI projects. On the other hand, LGD financing will push up the financing cost of GI projects. The interest rate of municipal investment bonds issued by financing platforms usually pushes up the market’s risk-free rate [32], and bank loans often use it as an important reference for loan rates [33], which forces GI investments to push up the financing cost to obtain bank loans. The crowding out of financing demand and the increase in financing cost will exacerbate the financing constraint problem and force a reduction in the financing demand, which in turn inhibits GI projects. Based on this, the following hypothesis is proposed.

**H2.**  *GI mediates the role of LGD and GTFP.*

Furthermore, we investigate whether raising the level of FMD in cities will lessen the detrimental effects of rising LGD on GTFP. First, the further development of the financial market would improve financial resource mismatch [34] and alleviate the financing constraint problem, thus mitigating the inhibitory influence of LGD on GTFP. Bank credit serves as the primary source of funding for local governments and businesses in China [35]. Credit discrimination is a constant problem for private sectors, and local governments can squeeze out private enterprise investment, resulting in financial resource mismatch and GTFP loss [34]. Along with the increase in the level of FMD, financial regulation will be strengthened, which will reduce credit discrimination in the private sector, enhance the effectiveness of credit fund allocation, and reduce GTFP losses. Second, increased FMD will intensify the competition among banks [36]. More bank competition will lead to an increase in credit supply [37], which would lessen the negative impact of LGD on GTFP. In addition, the banking industry’s increased competition will give the demand side of lending more negotiating power, which may secure longer financing terms and lower financing costs, further mitigating the negative effects. Based on the above analysis, the following hypotheses are proposed.

**H3.**  *The adverse effects of LGD on GTFP can be mitigated by the growth of FMD.*

## 3. Empirical Model and Methodology

### 3.1. The Calculation Method of GTFP

Referring to Tone (2001) [38], a production possibility set including both desired and non-desired outputs is established, and *k* cities in China are used as production decision units (DMUs), and each production unit contains three factor sets, which are the input factor set, the desirable (good) output set, and the non-desirable (bad) output set. For each DMU, it is assumed that *N* input factor indicators need to be used, and finally, *M* desired outputs are produced, as well as *I* non-desired outputs are produced.

Then, the SBM model considering the undesired output (environmental pollution) can be expressed as follows:

(1) $$min\rho^{*}=\frac{1-\frac{1}{N}\sum_{n=1}^{N}\frac{S_{n}^{-}}{x_{nk}}}{1+\frac{1}{M+I}\left(\sum_{m=1}^{M}\frac{S_{m}^{g}}{y_{mk}^{g}}+\sum_{i=1}^{I}\frac{S_{i}^{b}}{y_{ik}^{b}}\right)}$$

(2) $$s.t.\{\begin{array}{c} \begin{array}{c} x_{k}=X\lambda +S_{n}^{-}\\ y_{k}^{g}=Y^{g}\lambda -S_{m}^{g} \end{array}\\ \begin{array}{c} y_{k}^{b}=Y^{b}\lambda +S_{i}^{b}\\ S_{n}^{-}\geq 0,\;\;S_{m}^{g}\geq 0,\;\;S_{i}^{b}\geq 0,\;\;\lambda \geq 0\; \end{array} \end{array}$$

where N, M*,* and I denote the number of input indicators, desired output indicators, and non-desired output indicators, respectively; $S_{n}^{-}$, $S_{m}^{g}$, and $S_{i}^{b}$ represent the slack variables of factor inputs n, desired output m, and non-desired output i, respectively; X, $Y^{g}$, and $Y^{b}$ are the vector matrices corresponding to the input, desired output, and non-desired output values; $x_{k}$, $y_{k}^{g}$, and $y_{k}^{b}$ represent the input sets, the desired output sets, and the undesired output sets of the DMU *k*, respectively; similarly, $x_{nk}$, $y_{mk}^{g}$, and $y_{ik}^{b}$ represent the input *n*, the desired output *m*, and the undesired output *i* of the DMU *k*, respectively; *λ* is the weight vector; and $\rho^{*}$ is the efficiency value of the evaluation unit.

Since the SBM model cannot further evaluate and rank multiple decision units in the frontier plane, Tone (2002) [39] improved the SBM model and proposed the Super-SBM (Super-Efficient SBM) model, which is constructed as follows:

(3) $$min\rho^{**}=\frac{\frac{1}{N}\sum_{n=1}^{N}\frac{\overset{ˉ}{x}}{x_{nk}}}{\frac{1}{M+I}\left(\sum_{m=1}^{M}\frac{s_{m}^{g}}{y_{mk}^{g}}+\sum_{i=1}^{I}\frac{s_{i}^{b}}{y_{ik}^{b}}\right)}$$

(4) $$s.t.\{\begin{array}{c} \begin{array}{c} \bar{x}\geq X\lambda \\ {\bar{y}}^{g}\leq Y^{g}\lambda \end{array}\\ \begin{array}{c} {\bar{y}}^{b}\geq Y^{b}\lambda \\ \bar{x}\geq 0,\;{\bar{y}}^{g}\geq 0,\;{\bar{y}}^{b}\geq 0,\;\;\lambda \geq 0\; \end{array} \end{array}$$

where $\rho^{**}$ is the objective function value, which can be greater than or equal to 1, so that the evaluation and ranking problems of multiple decision units can be solved simultaneously and effectively. $\bar{x}$, ${\bar{y}}^{g}$, and ${\bar{y}}^{b}$ are the mean vectors for the inputs, the desired outputs, and the undesired outputs, respectively.

### 3.2. Econometric Models

#### 3.2.1. Basic Regression Model

Based on the previous theoretical analysis to estimate the impact of LGD on *GTFP* in China (Hypothesis 1), we first constructed fixed-effects model as follows:

(5) $$GTFP_{i,t}=\alpha_{0}+\alpha_{1}LGD_{i,t}+\sum^{\;}control_{i,t}+\mu_{i}+\sigma_{t}+\varepsilon_{i,t}$$

where $GTFP_{i,t}$ indicates the GTFP in city i in year t; $LGD_{i,t}$ indicates LGD scale in city i in year t; $control_{i,t}$ indicates a series of control variables; $\mu_{i}$ indicates city fixed effects; $\sigma_{t}$ indicates year fixed effect; and $\varepsilon_{i,t}$ indicates a random error term.

#### 3.2.2. Mediating Effect Model

To identify the mechanism of the role of *LGD* in inhibiting urban *GTFP* (Hypothesis 2), we constructed the mediating effect model, as follows, referring to the literature [40,41,42]:

(6) $$GI_{i,t}=\beta_{0}+\beta_{1}LGD_{i,t}+\sum^{\;}control_{i,t}+\mu_{i}+\sigma_{t}+\varepsilon_{i,t}$$

(7) $$GTFP_{i,t}=\gamma_{0}+\gamma_{1}LGD_{i,t}+\gamma_{2}GI_{i,t}+\sum^{\;}control_{i,t}+\mu_{i}+\sigma_{t}+\varepsilon_{i,t}$$

where $GI_{i,t}$ represents *GI* in city i in year t. This paper focuses on the coefficients $\beta_{1}$ and $\gamma_{2}$ to determine the presence or absence of mediating effect.

#### 3.2.3. Moderating Effect Model

Referring to the literature [43], we used the interaction term FMD×LGD  to identify the moderating effect of *FMD* (Hypothesis 3). Then, we incorporated the interaction term into the baseline model (5) to investigate whether there is a moderating effect. If the coefficient $\theta_{2}$ is significant, it indicates the presence of a moderating effect.

(8) $$GTFP_{i,t}=\theta_{0}+\theta_{1}LGD_{i,t}+\theta_{2}LGD_{i,t}\times FMD_{i,t}+\sum^{\;}control_{i,t}+\mu_{i}+\sigma_{t}+\varepsilon_{i,t}$$

where $FMD_{i,t}$ represents the level of FMD in city i in year t.

## 4. Definitions of Variable and Data Sources

### 4.1. Dependent Variable

Distinct from the traditional *TFP* calculation, *GTFP* contains two important connotations: improving productivity and protecting the environment [44]. In selecting input indicators, both productive factor inputs, such as human and capital inputs, and resource consumption, such as water supply and electricity supply, are selected and included as input variables. In the selection of output indicators, the maximization of desired outputs such as economic development and green ecological benefits are considered, as well as the constraints on economic development from three types of non-desired environmental pollution outputs, such as sewage, sulfur dioxide, and solid waste. Table 1 shows the selected input/output indicators. In terms of the choice of method, this paper adopts a non-radial, non-angle Super-SBM model to calculate *GTFP*. Referring to the literature [45], the input–output indicators of Super-SBM are shown in Table 1.

### 4.2. Independent Variable

According to the study of Huang et al. (2020) [9], we used the debt balance of each city’s financing platform company as a share of GDP to indicate the level of *LGD.*

### 4.3. Mediating Variable

In the Hypothesis 2, the mediating variable is the *GI*. According to the study of Tolliver et al. (2021) [46], we measured the *GI* in each city by the natural logarithm value of the amount of green invention patent applications.

### 4.4. Moderating Variable

In the Hypothesis 3, the moderating variable is the *FMD*. According to the study of Chen (2018) [47], this paper measured the level of the *FMD* of each city using the percentage of financial institutions’ loan balance to GDP at year-end.

### 4.5. Controlled Variables

This paper controls for a vector of city-level control variables. The economic development (*PGDP*), the level of economic development, reflects the growth in *TFP*, which is a part of GTFP. An upgrade in industrial structure (*INS*) will lead to the transfer of resources, eliminate high pollution and high emission enterprises, and promote GTFP [48]. The level of foreign direct investment (*FDI*), according to international trade theory, on the one hand, can bring advanced industries and technology spillover and promote GTFP; on the other hand, it can also trigger the “pollution haven” effect and suppress GTFP [49]. The intensity of environmental regulation (*ER*), according to the Porter effect hypothesis, can promote GTFP through technological innovation [50]. The population size (*POP*): the larger the population, the greater the environmental pressure, which has a negative impact on GTFP [51]. The infrastructure (*INFRA*): an improvement of infrastructure may accelerate the flow of production factors and promote GTFP [52].

Following the literature on green productivity, the variable *PGDP* is indicated by the natural logarithm of GDP per capita [53]; the variable *INS* is represented by the share of tertiary industry in GDP [3]; the variable *FDI* is measured by the share of regional foreign direct investment in GDP [54]; the variable *ER* is represented by the share of total investment in environmental pollution control in GDP [55]; the variable *POP* is measured by the natural logarithm value of population size; and the variable *INFRA* is represented by the per capita area of roads [54]. Table 2 displays the descriptive statistics for the variables.

### 4.6. Data Sources and Statistical Characteristics

The China City Statistical Yearbook (China City Statistical Yearbook data are from the Chinese National Bureau of Statistics website (https://data.stats.gov.cn/easyquery.htm?cn=C01) (accessed on 1 January 2020)), the Wind database, and the State Intellectual Property Office’s patent search system were primarily used in this study to gather city-level economic and social statistics, as well as information on LGD and green patents. The research period for this work is 2010–2019, and because of the severe absence of data on pertinent variables specific to individual cities, 271 cities were ultimately chosen as the research sample based on the standards of data accessibility and validity, covering 31 provincial-level administrative regions, representing more than 90% of China’s total number of cities.

## 5. Empirical Results

### 5.1. Baseline Regression

To verify the impact of LGD expansion on GTFP, regressions were conducted according to Model 5. Table 3 reports the results of the baseline regression. The explanatory variable is LGD to GDP (LGD), column (1) is the results of the OLS regression, column (2) adds control variables to column (1), and column (3) further controls for area-fixed and time-fixed effects. In the regression results, the regression coefficients of the core explanatory variables were all significantly negative at the 1% level, indicating that the expansion of LGD significantly reduces the growth rate of GTFP. Hypothesis 1 was tested. Each standard deviation increase in the level of LGD decreased the level of urban GTFP by 0.51% (0.035 × 0.147). In the regression results of the control variables, there was a significant positive relationship between the level of economic development, industrial structure, environmental regulation intensity, and GTFP. This indicates that the level of GTFP will increase with economic development, upgrades in industrial structure, and intensity of environmental regulation. Population size was significantly negatively correlated with GTFP, indicating that population size suppresses the level of GTFP.

### 5.2. Robustness Tests

To ensure that the results of this paper are more credible, we conducted extensive robustness tests. The results are shown in Table 4. First, this paper adopted the method of Shi and Li (2019) [56], which replaces the original non-desired output indicator with CO_2_ emissions and re-measures GTFP. As seen in column (1), the regression results of replacing GTFP were still significantly negative at the 1% level. Second, the explanatory variables were replaced. Referring to the existing literature [9], the natural logarithm of the debt balance of the financing platform company (LnLGD) was used as a proxy variable for LGD financing. Column (2) shows that the regression coefficient was still significantly negative at the 5% level. Third, to alleviate the possible endogeneity problem, this paper conducted a regression analysis using LGD data with a one-period lag. As seen in column (3), the results remained robust. Finally, since Beijing, Shanghai, Tianjin, and Chongqing are municipalities directly under the central government, they have more advantages in terms of political, economic, and other resources compared to other cities, which may affect the findings of the study. For this reason, the paper excludes four municipalities from the robustness test. In column (4), the regression results did not change substantially, and the rising level of LGD had a significant negative effect on GTFP. In summary, the conclusion that LGD inhibits GTFP is robust.

### 5.3. Mediating Effects

The results of the previous benchmark regressions show that the net effect of LGD expansion on GTFP is negative. In the theoretical analysis, LGD expansion inhibits GTFP growth by reducing the level of GI in cities. Based on the results of the benchmark regression, this paper tests the impact mechanism, i.e., to verify Hypothesis 2. The test was divided into three steps based on the principle of the mediating effect. The first step was completed in the baseline regression, and the second step was to test the mechanism variable (at this point as the explanatory variable) and civilized city selection (at this point, as the explanatory variable). The third step was to regress the explanatory variable, urban investment debt, on the core explanatory variable, civilized city selection, and the mechanism variable.

Columns (1) and (3) of Table 5 report the regression results for GI as mediating variables, respectively. It can be seen from columns (1) and (3) that the expansion of LGD significantly reduces the level of GI. Furthermore, the regression results in column (2) show that the coefficients of GI and LGD are 0.032 and −0.016, respectively, and both pass the significance test at the 5% level at least. It is thus clear that LGD expansion inhibits urban GTFP by reducing the level of GI. Hypothesis 2 was tested.

### 5.4. Moderating Effects

To test whether the level of FMD has a moderating influence on the relationship between LGD and GTFP, we used the moderated regression approach model (8). Column (1) in Table 6 uses the indicator as a proxy for the level of FMD. The regression coefficient for the interaction terms *LGD × FMD* is shown to be 0.018 and significant at the 5% level. This shows that the inhibiting effect of LGD expansion on GTFP is lessened as FMD increases. That is, the level of FMD plays a significant positive moderating effect between LGD expansion and GTFP.

### 5.5. Heterogeneity Analysis

This study first divided the sample into three subsamples based on geographic location: eastern region, central region, and western region for a regression analysis. (Appendix A) The results are displayed in columns (1)–(3) of Table 7. The heterogeneity of different regions may affect the inhibitory effect of LGD financing on GTFP. The main explanatory variables’ regression coefficient in the eastern region is negative but not statistically significant, as shown; the central region’s regression coefficient, which measures significance at the 5% level, is −0.040; and the western region’s regression coefficient is −0.045, which is considerably negative at the 1% level. This may be since the more economically developed cities are more tolerant of LGD. Second, this paper conducts a heterogeneity analysis by dividing the sample into two categories of high and low debt levels according to the mean value of the proportion of LGD to GDP. In columns (4) and (5) of Table 7, the detrimental effects of LGD expansion on GTFP are more prominent in cities with higher government debt levels, and the absolute value of the coefficient is larger. In contrast, in cities with lower government debt levels, the extent of the above negative impact is weakened, and the absolute value of the coefficient is smaller and insignificant. 

## 6. Conclusions and Policy Implications

Green development has recently taken center stage in China’s economy. This study explored the effect of LGD financing on GTFP based on data from 271 Chinese cities between 2010 and 2019. The following are the primary conclusions: First, the GTFP of cities decreases as LGD increases. In China’s central and western areas, where cities have higher debt levels, this negative correlation is particularly pronounced. Second, LGD decreases urban GTFP by lowering the amount of urban GI, and GI is a mediating factor. Finally, the environmental deterrent effect of LGD is significantly moderated by the increase in development in urban financial markets, i.e., FMD aids in mitigating the detrimental effect of LGD on urban GTFP.

The recommendations made in this study are based on the previous findings. First, given that Chinese LGD might hinder urban GI and inhibit GTFP, to foster long-term growth of China’s green economy, the government should focus on mitigating risks associated with LGD, preventing its heedless expansion, enhancing its governance of LGD, and putting in place a reliable LGD financing system. Second, FMD can effectively mitigate the detrimental impact of LGD on GTFP. Chinese authorities should accelerate the reform of the financial market, optimize the financial market environment, correct the distortion of financial resources, and guide the capital “out of government and into the real world” to promote economic growth; on the other hand, they should encourage green finance, green credit, and other innovative models to support the development of China’s low-carbon transition. Finally, local governments have a greater impact on the suppression of GTFP in less-developed cities and cities with higher debt levels, which generally have lower debt repayment and governance capacity. Therefore, in specific government debt governance measures, the Chinese government should take into account the heterogeneous impacts of cities in different regions and debt levels, implement differentiated policies, and increase environmental governance characteristic indicators to reduce debt risks while promoting coordinated and sustainable development of environmental protection and the economy.

## Figures and Tables

**Table 1:** Definition of input and output metrics for *GTFP* calculations.

First-Level Indicators	Second-Level Indicators	Definition
Inputs	Workforce	The total amount of employment in urban units at year-end
	Capital Stock	Estimated by the perpetual inventory method
	Water Supply	Annual water supply
	Electricity Supply	Annual power generation
Desirable outputs	GDP	Real gross domestic product measured in 2000 as the base period
	Green Coverage	The percentage of built-up area that is covered by forest
Undesirable outputs	Industrial Waste	The quantity of industrial wastewater dumped
	Sulfur Dioxide Emissions	The quantity of industrial sulfur dioxide emitted
	Soot Emission	The quantity of industrial soot (dust) emitted

**Table 2:** Descriptive statistics.

Variables Type	Variables	Obs.	Mean	Std. Dev.	Min	Max
Dependent Variable	*GTFP*	2710	1.010	0.029	0.960	1.067
Independent Variable	*LGD*	2710	0.129	0.147	0.000	0.984
Mediating Variable	*GI*	2710	3.784	1.948	0.000	10.182
Moderating Variable	*FMD*	2710	0.978	0.636	0.118	9.623
Controlled Variables	*PGDP*	2710	10.580	0.611	8.553	12.281
	*INS*	2710	0.408	0.102	0.098	0.835
	*FDI*	2710	0.028	0.027	0.000	0.194
	*ER*	2710	0.889	0.125	0.001	1.501
	*POP*	2710	5.707	0.863	2.356	8.137
	*INFRA*	2710	16.927	7.269	1.419	60.070

**Table 3:** Effect of LGD on GTFP.

	(1)	(2)	(3)
	GTFP	GTFP	GTFP
LGD	−0.106 ***	−0.043 ***	−0.035 ***
	(0.007)	(0.010)	(0.011)
PGDP		0.210 ***	0.189 ***
		(0.044)	(0.058)
IS		0.844 ***	0.825 ***
		(0.133)	(0.239)
FDI		0.528 **	0.434
		(0.211)	(0.319)
ER		0.450 ***	0.303 ***
		(0.071)	(0.097)
POP		−0.108 ***	−0.089 **
		(0.029)	(0.041)
INFRA		0.028	0.005
		(0.037)	(0.045)
Cons	1.024 ***	−1.823 ***	0.950 ***
	(0.021)	(0.016)	(0.111)
N	2710	2710	2710
Year FE	No	No	Yes
City FE	No	No	Yes
Adj. R^2^	0.093	0.132	0.155

Note: Robustness standard errors are in parentheses, ***, **, * denote passing the test at 1%, 5%, and 10% significance levels, respectively.

**Table 4:** Robustness test.

	(1)	(2)	(3)	(4)
	Alternative GTFP	GTFP	GTFP	GTFP
LGD	−0.112 ***			−0.041 ***
	(0.032)			(0.012)
LnLGD		−0.227 **		
		(0.099)		
L. LGD			−0.041 ***	
			(0.013)	
Cons	0.491 ***	0.932 ***	1.069 ***	0.958 ***
	(0.003)	(0.111)	(0.137)	(0.111)
N	2710	2710	2710	2670
Controls	Yes	Yes	Yes	Yes
Year FE	Yes	Yes	Yes	Yes
City FE	Yes	Yes	Yes	Yes
Adj. R^2^	0.226	0.158	0.168	0.156

Note: Robustness standard errors are in parentheses, ***, **, * denote passing the test at 1%, 5%, and 10% significance levels, respectively.

**Table 5:** Mediating effects test.

	(1)	(2)
	GI	GTFP
LGD	−0.611 ***	−0.016 **
	(0.178)	(0.07)
GI		0.032 ***
		(0.010)
Cons	−5.899 ***	0.948 ***
	(1.610)	(0.112)
N	2710	2710
Controls	Yes	Yes
Year FE	Yes	Yes
City FE	Yes	Yes
Adj. R^2^	0.679	0.256

Note: Robustness standard errors are in parentheses, ***, **, * denote passing the test at 1%, 5%, and 10% significance levels, respectively.

**Table 6:** Moderating effects test.

	(1)
	GTFP
LGD	−0.048 *
	(0.026)
LGD × FMD	0.018 **
	(0.009)
Cons	0.944 ***
	(0.111)
N	2710
Controls	Yes
Year FE	Yes
City FE	Yes
Adj. R^2^	0.164

Note: Robustness standard errors are in parentheses, ***, **, * denote passing the test at 1%, 5%, and 10% significance levels, respectively.

**Table 7:** Heterogeneity analysis.

	(1)	(2)	(3)	(4)	(5)
	East	Central	Western	High	Low
LGD	−0.028	−0.040 **	−0.045 ***	−0.078 ***	−0.020
	(0.015)	(0.017)	(0.012)	(0.028)	(0.020)
Cons	0.688 **	1.362 ***	0.900 **	0.982 ***	0.487 *
	(0.288)	(0.217)	(0.414)	(0.140)	(0.286)
N	990	970	750	1640	1070
Controls	Yes	Yes	Yes	Controls	Yes
Year FE	Yes	Yes	Yes	Yes	Yes
City FE	Yes	Yes	Yes	Yes	Yes
Adj. R^2^	0.147	0.118	0.202	0.211	0.259

Note: Robustness standard errors are in parentheses, ***, **, * denote passing the test at 1%, 5%, and 10% significance levels, respectively.

## Data Availability

The data in this study are available from the authors upon request.

## Appendix A

In Section 5.5 Heterogeneity analysis, our sample is divided into three subsamples based on geographical location: eastern, central, and western regions. Among them, the specific provinces in the three subsamples are listed below.

**Table A1:** China’s provincial administrative divisions (eastern, central, and western regions).

Eastern Region	Central Region	Western Region
Beijing, Tianjin, Hebei, Liaoning, Shanghai, Jiangsu, Zhejiang, Fujian, Shandong, Guangdong, Hainan; Total 11 provinces.	Shanxi, Neimenggu, Jilin, Heilongjiang, Anhui, Jiangxi, Henan, Hubei, Hunan, Guangxi; Total 10 provinces.	Sichuan, Guizhou, Yunnan, Xizang, Shaanxi, Gansu, Qinghai, Ningxia, Xinjiang; Total 9 provinces.

We list all the abbreviations in the full text in the table below.

**Table A2:** Abbreviations and full name.

Variables Type	Variables	Full Name
Dependent Variable	GTFP	Green total factor productivity
Independent Variable	LGD	Local government debt
Mediating Variable	GI	Green innovation
Moderating Variable	FMD	Financial market development
Controlled Variables	PGDP	Per capital GDP
	INS	Industrial structure
	FDI	Foreign direct investment
	ER	Environment regulation
	POP	Population size
	INFRA	Infrastructure
Reporting in Regression Models	Cons	Intercept term in the regression
	N	Number of observations
	Year FE	Whether the year is fixed in the fixed effects model
	City FE	Whether the city is fixed in the fixed effects model
	Adj. R^2^	Adjusted R-Squared
	Controls	Whether control variables are added
